# Stochastic Neuromorphic Computing Architecture Based on Voltage-Controlled Probabilistic Switching Magnetic Tunnel Junction (MTJ) Devices

**DOI:** 10.3390/mi17020216

**Published:** 2026-02-05

**Authors:** Liang Gao, Chenxi Wang, Yanfeng Jiang

**Affiliations:** 1 School of Integrated Circuits, Jiangnan University, Wuxi 214122, China; 6231916022@stu.jiangnan.edu.cn; 2 Institute of Computing Technology, Chinese Academy of Sciences, Beijing 100190, China; wangchenxi@ict.ac.cn

**Keywords:** Voltage-Controlled Magnetic Anisotropy, Spin Hall Effect, Magnetic Tunnel Junction, stochastic switching, in-memory computing, Binarized Neural Network

## Abstract

As integrated circuits face increasingly stringent demands regarding power consumption, area, and stability, integrating novel spintronic devices with computing architectures has become a crucial direction for breaking through traditional computing paradigms. In the paper, switching mechanism of Magnetic Tunnel Junctions (MTJs) under the synergistic effect of Voltage-Controlled Magnetic Anisotropy (VCMA) and the Spin Hall Effect (SHE) is investigated. VCMA-assisted switching SHE-MTJ device is adopted, and a macrospin approximation model is established based on the Landau-Lifshitz-Gilbert (LLG) equation to systematically analyze its dynamic characteristics. The research demonstrates that applying VCMA voltage pulses with appropriate amplitude and width can significantly reduce the required spin Hall current density and pulse width for switching, thereby effectively minimizing ohmic losses and Joule heating. Furthermore, by incorporating a thermal fluctuation field, voltage-controlled SHE-MTJ device with stochastic switching behavior can be constructed, obtaining an approximately sigmoidal voltage-probability response curve. This provides an ideal physical foundation for stochastic computing and neuromorphic computing. Based on the above established fundamental discovery, an in-memory computing architecture supporting binarized Convolutional Neural Networks (CNNs) is proposed and designed in the paper. Combined with the lightweight network SqueezeNet, this architecture achieves a Top-1 recognition accuracy of 72.49% on the CIFAR-10 dataset, with a parameter count of only 1.25 × 10^6^. This work offers a feasible spintronic implementation scheme for low-power, high-energy-efficiency edge-side intelligent chips.

## 1. Introduction

With the rapid development of artificial intelligence, Internet of Things (IoTs), and edge computing, unprecedented demands have been placed on the energy efficiency, speed, and integration density of computing systems [1]. The traditional von Neumann architecture suffers from the “memory wall” problem, where frequent data movement between physically separated computing and memory units leads to high energy consumption and time latency [2]. This has become a key bottleneck limiting the performance of modern computing systems. To overcome this limitation, in-memory computing has emerged as a revolutionary architectural paradigm. Its core idea is to embed computational functionality within memory cell arrays, performing operations directly at the data storage location, thereby minimizing data movement [3].

Among various potential physical carriers for in-memory computing, spintronic devices have attracted considerable attention due to their non-volatility, high speed, ultra-high endurance, and excellent scalability. Magnetic Tunnel Junction (MTJ), a core device in spintronics, characterizes its resistance state based on the relative orientation (parallel P or anti-parallel AP) of the free layer magnetization [4,5,6,7]. MTJs have been successfully applied in Magnetic Random-Access Memory (MRAM). Not only can MTJs serve as ideal memory cells, but their inherent physical properties also make them promising candidates for implementing analog and logic computing functions.

The write energy (magnetization switching energy) of MTJs is a critical factor determining their application prospects. Early field-driven switching methods have been phased out due to high power consumption and poor scalability [8,9]. Spin–Transfer Torque (STT) mechanism utilizes a polarized current flowing through the MTJ to directly switch the magnetization, significantly reducing the write current. However, it still faces reliability challenges from the shared read/write current path, and there remains room for further reduction in energy consumption [10,11,12,13]. Spin–Orbit Torque (SOT) mechanism manipulates magnetization by generating spin current via charge current in an adjacent heavy metal (HM) layer, achieving separated read/write paths, improving device reliability, and exhibiting faster switching speeds. Nevertheless, to overcome the high energy barrier in Perpendicular Magnetic Anisotropy (PMA) MTJs, the required SOT critical current density remains relatively high (typically >10^11^ A/m^2^), leading to significant Joule heating and power consumption [14,15,16].

To further reduce switching energy, Voltage-Controlled Magnetic Anisotropy (VCMA) effect has been introduced as an auxiliary means [17,18,19]. The VCMA effect originates from the modulation of electron orbital occupancy at the ferromagnet/oxide interface by an electric field, thereby altering the interfacial magnetic anisotropy energy. Its primary advantage lies in dynamically adjusting the MTJ’s energy barrier by applying only a voltage (not current), theoretically enabling ultra-low energy switching approaching the thermodynamic limit. Combining VCMA with SOT to construct a VCMA-SOT hybrid writing scheme has become one of the most promising technological paths for reducing MTJ write energy [17]. Studies have shown that VCMA voltage can effectively reduce the critical current and pulse width required for SOT switching. However, existing research has predominantly focused on deterministic switching characteristics. There is a lack of in-depth exploration regarding the probabilistic switching behavior of VCMA-SOT-MTJs under thermal disturbances and its systematic modeling [19]. This unique characteristic can be used for building novel stochastic computing units and bio-inspired neuromorphic devices.

On the other hand, with the remarkable success of neural networks (particularly Convolutional Neural Networks (CNNs)) in visual tasks, a pressing challenge arises: how to efficiently deploy these computationally intensive models on resource-constrained edge devices. Model lightweighting (e.g., network pruning, quantization) and specialized hardware acceleration are two mainstream directions [20]. Among them, Binarized Neural Networks (BNNs) constrain weights and activations to +1/−1, replacing expensive floating-point multiply-accumulate operations with efficient XNOR and bit-count operations, greatly reducing computational and memory overhead [21,22]. Simultaneously, incorporating inherent randomness from biological neural systems to enhance model generalization and robustness has become an emerging perspective in neural network design [23]. Utilizing physical devices with inherent randomness (e.g., stochastically switching MTJs) as fundamental computing units for neural networks, realizing “physics-inspired computing,” opens a new avenue for constructing ultra-low-power, highly parallel in-memory computing systems.

Based on the above background, this study aims to deeply integrate the device physics advantages of VCMA-SOT-MTJs with the computational demands of neural networks, conducting systematic research from the underlying device to the top-level architecture. In the paper, an accurate macro-spin model of the device is firstly established, thoroughly analyzing the regulatory effect of VCMA voltage on the deterministic switching dynamics of SOT-driven MTJs, and quantifying its efficacy in reducing critical current and shortening pulse width. Subsequently, the impact of thermal fluctuations on the switching process is introduced and simulated innovatively, extending the deterministic switching device into a voltage-controlled probabilistic switching device. A highly controllable and regularly shaped sigmoidal voltage-switching probability curve can be obtained, which establishes the physical foundation for constructing genuine stochastic computing units and neurons with stochastic activation functions.

Finally, based on this voltage-controlled stochastic switching SHE-MTJ device, an in-memory computing architecture suitable for binarized convolutional neural networks (CNNs) is designed. In the architecture, the MTJ crossbar arrays are utilized to store the binarized weights in situ, and naturally analog multiply-accumulate results are converted into VCMA probability control voltages for driving the next layer of neurons through a resistive voltage divider network. Thus, information storage, logical computation, and stochastic nonlinear activation are synchronously accomplished within a single compact structure. This scheme is instanced on the classic lightweight network SqueezeNet and validated its feasibility on the CIFAR-10 dataset. This research not only provides new ideas at the device and circuit levels for developing ultra-low-energy spintronic in-memory computing chips but also offers a valuable hardware implementation scheme for stochastic computing and neuromorphic computing systems targeting edge intelligence.

## 2. VCMA-Assisted Switching SHE-MTJ

### 2.1. Theoretical Model of SHE-MTJ Assisted by VCMA

**Figure 1:**
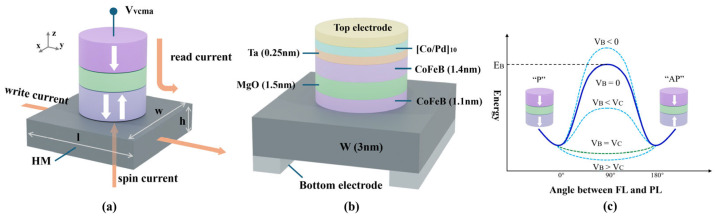
Schematic of the SHE-MTJ device structure and the VCMA effect. (**a**) Schematic of the SHE-MTJ with separated read/write paths; (**b**) Layer stack of the device; (**c**) Illustration of the MTJ energy barrier modulation under VCMA voltage.

Voltage-Controlled Magnetic Anisotropy (VCMA) is a method to modulate magnetic anisotropy or achieve magnetization switching by applying an electric field. Its physical mechanism can be explained as electric-field-induced interfacial charge accumulation, which alters the orbital occupancy of interfacial atoms, thereby modulating magnetic anisotropy through spin–orbit coupling. By avoiding current-based control, VCMA can significantly reduce ohmic losses and Joule heating, thereby substantially decreasing MTJ switching energy. VCMA can assist both STT and SOT for MRAM writing. This writing approach can effectively reduce the critical switching current density. At the same time, precessional magnetization switching can be enabled directly by applying voltage pulses via VCMA. While VCMA is used on SOT-MRAM, its operation diagram is shown in Figure 1a.

MTJs with a CoFeB/MgO/CoFeB structure [24,25] possess high Perpendicular Magnetic Anisotropy (PMA) and high Tunnel Magnetoresistance (TMR), making them the core devices widely used in current MRAM, as shown in Figure 1b. The PMA at the ferromagnet/oxide interface can be modulated by an external voltage (or electric field), with a positive voltage reducing PMA, while a negative voltage improving it. The “P” and “AP” states of the MTJ are separated by an energy barrier E_B_. The thermal stability factor Δ describes its resistance to disturbances. Under the influence of the VCMA effect, E_B_ can be expressed as:

(1) $$E_{B}\left(V_{B}\right)\approx \left[K_{S}\left(V_{B}\right)-2\pi M_{S}^{2}\left(N_{Z}-N_{X,Y}\right)t_{f}\right]\cdot A,$$

where $K_{S}\left(V_{B}\right)$ is the voltage-modulated interfacial anisotropy energy density, $V_{B}$ is the voltage applied across the insulating layer, $M_{S}$ is the saturation magnetization, $N_{Z}$ and $N_{X,Y}$ are the longitudinal and transverse demagnetization factors, $t_{f}$ is the free layer thickness, and A is the cross-sectional area of the MTJ [26].

$K_{S}\left(V_{B}\right)$ can be expressed as a linear function of $V_{B}$:

(2) $$K_{S}\left(V_{B}\right)=K_{S}\left(0\right)-\kappa V_{B}/t_{\mathrm{ox}},$$

where $K_{S}\left(0\right)$ is the interfacial PMA at zero voltage, and κ is the voltage-controlled anisotropy coefficient. Substituting Equation (2) into Equation (1) yields the voltage-dependent thermal stability factor:

(3) $$\Delta \left(V_{B}\right)=E_{B}\left(V_{B}\right)/k_{B}T=\Delta \left(0\right)-\xi AV_{B}/k_{B}Tt_{ox},$$

where $\Delta \left(0\right)$ is the thermal stability factor at zero voltage, $k_{B}$ is the Boltzmann constant, and T is the temperature. Setting $\Delta \left(V_{B}\right)$ = 0 defines the critical voltage $V_{C}$, which is the minimum voltage required to completely eliminate the MTJ’s energy barrier:

(4) $$V_{C}=\Delta \left(0\right)k_{B}Tt_{ox}/\xi A,$$

If $E_{B}$ is sufficiently large, the two states remain thermally stable. When switching the MTJ magnetization, applying a VCMA voltage $V_{B}$ can reduce or even eliminate the switching barrier, as shown in Figure 1c. When the applied $V_{B}$ exceeds the critical voltage $V_{C}$, the energy barrier is completely eliminated, and the magnetization state of the MTJ free layer becomes unstable, oscillating between “P” and “AP” states, entering a precessional switching regime. When 0 < $V_{B}$ < $V_{C}$ is applied, the barrier is reduced but not completely eliminated. Thermal fluctuations may cause the MTJ free layer magnetization to switch or return to its initial state under the damping torque, corresponding to a thermally activated switching regime [5,27]. In practical applications, the precessional regime requires precise control of the voltage pulse width, while the thermally activated regime typically requires assistance from an external magnetic field or charge current for switching.

Figure 1a shows a schematic of the voltage-controlled SHE-MTJ structure proposed in this study. When a write current flows along the y-axis through the heavy metal (HM) layer, it generates a spin current in the perpendicular direction, driving oscillations or switching of the magnetization in the adjacent MTJ free layer [26,27]. The read current flows vertically through the MTJ, using a small sensing current to detect its resistance state. The device shown in Figure 1b is a typical CoFeB/MgO/CoFeB sandwich structure, with W as the HM layer material. Two electrodes are placed at the bottom for the write current, and one electrode is at the top for applying the read current and VCMA voltage [28,29].

With the write current along the y-direction in the HM layer, the MTJ magnetization dynamics can be described by the LLG equation including the SHE term [13]:

(5) $$\frac{dm}{dt}=-\gamma m\times H_{eff}+\alpha m\times \frac{dm}{dt}+\rho \eta J_{SHE}m\times \left(m\times \sigma_{SHE}\right),$$

where γ is the gyromagnetic ratio, $H_{eff}$ is the effective field, α is the damping coefficient, $\rho =\gamma \hbar /2e\mu_{0}t_{f}M_{S}$, where ℏ is the reduced Planck constant, e is the electron charge, $\mu_{0}$ is the vacuum permeability, η is the spin Hall angle of the material, $J_{SHE}$ is the transverse current density flowing through the HM layer, and $\sigma_{SHE}$ is the spin current polarization direction [29].

$H_{\mathrm{eff}}$ represents the vector sum of all effective fields present and can be expressed as:

(6) $$H_{\mathrm{eff}}=H_{external}+H_{demag}+H_{thermal}+H_{aniso}+\ldots ,$$

The demagnetization field $H_{\mathrm{demag}}$, describing the shape anisotropy of the free layer, is expressed as [30]:

(7) $$H_{\mathrm{demag}}{=-M}_{s}\left(N\cdot m\right),$$

where $N=\{N_{X},N_{Y},N_{Z}\}$ can be described using a simplified shape anisotropy model:

(8) $$N_{x}=0.5\left(1-N_{Z}\right),N_{Y}=0.5\left(1-N_{Z}\right),N_{Z}=\frac{\beta^{2}}{\beta^{2}-1}\left\{1-\frac{\sin^{-1}\left[{\left(\beta^{2}-1\right)}^{\frac{1}{2}}/\beta \right]}{{\left(\beta^{2}-1\right)}^{\frac{1}{2}}}\right\},$$

where $\beta =d/t_{f}$, and d is the diameter of the MTJ.

$H_{aniso}$ is the voltage-controlled magnetic anisotropy field originating from the interfacial PMA, expressed as:

(9) $$H_{aniso}=\frac{2\left(K_{S0}+\kappa V_{VCMA}/t_{ox}\right)}{\mu_{0}M_{S}t_{f}}m,$$

where $K_{s0}$ is the interfacial anisotropy energy density constant, κ is the voltage-controlled anisotropy coefficient, $V_{VCMA}$ is the VCMA voltage applied across the MTJ, and $t_{ox}$ is the insulator layer thickness.

When solving the LLG equation, $H_{\mathrm{eff}}$ is first decomposed into Cartesian components $H_{\mathrm{eff}}=H_{X}X+H_{Y}Y+H_{Z}Z$, and m is decomposed as $m=m_{x}x+m_{y}y+m_{z}z$. Then, the spherical coordinate system (r,θ,ψ) is used to describe m. With r = 1, m=(sinθcosψ,sinθsinψ,cosθ). For the SHE-MTJ without considering thermal fluctuations, $H_{\mathrm{eff}}=H_{external}+H_{demag}+H_{aniso}$ can be decomposed as:

(10) $$\begin{array}{l} H_{x}=H_{externalx}-N_{X}M_{S}\sin\theta \cos\varphi \\ H_{y}=H_{externaly}-N_{Y}M_{S}\sin\theta \sin\varphi \\ H_{z}=H_{externalz}-N_{Z}M_{S}\cos\theta +\frac{2\left(K_{S0}+\kappa V_{VCMA}/t_{ox}\right)}{\mu_{0}M_{S}t_{f}} \end{array},$$

Substituting the spherical coordinate representations of all variables into Equation (1) allows solving for the time-dependent spherical coordinate variables:

(11) $$\left\{\begin{array}{l} \frac{d\varphi }{dt}=\frac{\gamma }{\left(1+\alpha^{2}\right)\sin\theta }\left[H_{x}\left(-\cos\theta \cos\varphi -\alpha \sin\varphi \right)+H_{y}\left(-\cos\theta \sin\varphi +\alpha \sin\varphi \right)+H_{z}\sin\theta \right]+\frac{\rho \eta J_{SHE}}{\left(1+\alpha^{2}\right)\sin\theta }\left(\sin\varphi -\alpha \cos\varphi \right)\cos\theta \\ \frac{d\theta }{dt}=\frac{\gamma }{\left(1+\alpha^{2}\right)}\left[H_{x}\left(\alpha \cos\theta \cos\varphi -\sin\varphi \right)+H_{y}\left(\alpha \cos\theta \sin\varphi +\alpha \cos\varphi \right)-\alpha H_{z}\sin\theta \right]-\frac{\rho \eta J_{SHE}}{\left(1+\alpha^{2}\right)}\left(\cos\varphi \cos\theta +\alpha \sin\varphi \right) \end{array}\right.,$$

### 2.2. Simulation Methodology and Dynamic Switching Observation

Verilog-A model for MTJ device with a CoFeB/MgO/CoFeB structure is adopted, taking into account voltage-assisted spin Hall effect switching. Established within the framework of the macrospin Landau–Lifshitz–Gilbert–Slonczewski equation, the model integrates voltage-controlled magnetic anisotropy, spin–orbit coupling, and the spin Hall effect to investigate the specific impact of VCMA on spin–orbit torque switching. Simulation results demonstrate that the introduction of the VCMA effect significantly reduces the critical SOT current ($I_{SOT}$), enabling magnetization switching through the combination of a minimal ($I_{SOT}$) and a sufficient MTJ voltage ($V_{VCMA}$). Furthermore, a specific switching condition is identified that achieves the shortest switching path while maintaining ultra-low power consumption. Moreover, the application of a pulse writing scheme effectively enhances both the switching speed and reliability of the device.

**Figure 2:**
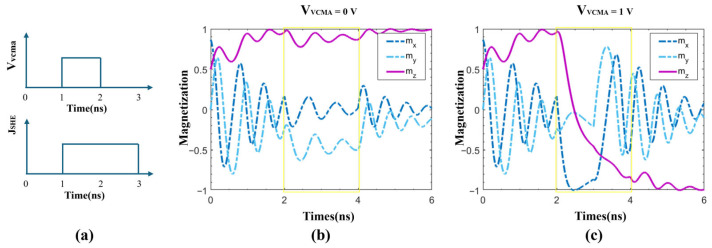
Modulation of magnetization switching dynamics in SHE-MTJ by VCMA voltage. (**a**) Timing of applied VCMA voltage and HM current pulses; (**b**) Time evolution of three magnetization components with VCMA = 0 V; (**c**) Time evolution of three magnetization components with VCMA = 1 V.

To facilitate observation of magnetization changes under voltage influence, during simulation, the initial polar angle of the MTJ free layer magnetization is set to π/3, and the azimuthal angle to 0. Figure 2 exhibits the modulation of magnetization switching dynamics in SHE-MTJ by VCMA voltage. As shown in Figure 2a, during 0–2 ns, no voltage or current is applied. The magnetization precesses under the external field and Gilbert damping. At 1 ns, a pulse current with a width of 2 ns and a density of $2.2\times 10^{11}A/M^{2}$ is applied to the HM layer (indicated by the yellow rectangle in the figure), simultaneously with a VCMA voltage pulse of 1 ns width applied to the MTJ. Figure 2b shows the case without VCMA voltage, where the SOT torque is insufficient to overcome damping, the MTJ fails to switch, and the magnetization returns to its initial state after the current stops. Figure 2c shows the result with a 1 V VCMA voltage applied. Here, the SOT torque exceeds the damping torque, the magnetization z-component deflects to −1, and after the current stops at 4 ns, $m_{Z}$ stabilizes at −1, indicating successful MTJ switching. The total simulation time is 10 ns. Since the three magnetization components stabilize after 6 ns, they are not plotted beyond that point.

**Figure 3:**
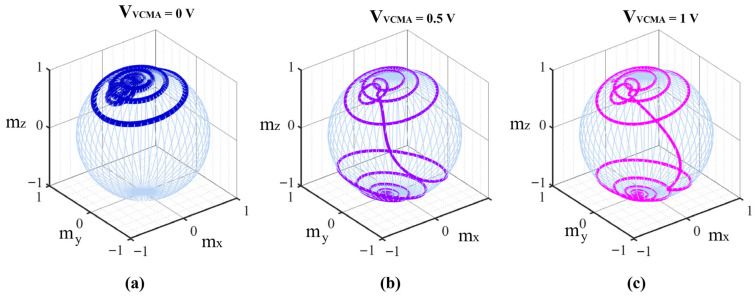
Precession trajectories of the free-layer magnetization under different VCMA voltages. (**a**) VCMA = 0 V; (**b**) VCMA = 0.5 V; (**c**) VCMA = 1.0 V.

To visually illustrate the magnetization evolution path, Figure 3 plots the spiral precession trajectories of the magnetization for VCMA voltages of 0 V, 0.5 V, and 1.0 V. It can be seen that without VCMA assistance, the magnetization oscillates and returns to its initial state. With VCMA voltage assistance, the magnetization spirals downward. After switching and removal of the external field, the magnetization oscillates and stabilizes at (0,0,−1).

The aforementioned simulation results vividly demonstrate the dynamic facilitation of SOT-driven magnetization switching by the VCMA effect. To move beyond qualitative observation and quantitatively assess the potential of this hybrid mechanism for practical device applications—particularly in reducing power consumption—a systematic investigation of the switching critical conditions is imperative. The following section presents parametric scans that quantify the impact of VCMA assistance on lowering the write current and shortening the pulse window.

### 2.3. Parametric Analysis and Optimization of Switching Critical Conditions

To quantify the benefit of VCMA assistance in reducing SOT write requirements, we systematically investigated the synergy between the SOT current density ($J_{SHE}$), pulse width ($t_{SHE}$), and the VCMA voltage amplitude ($V_{VCMA}$), pulse width ($t_{VCMA}$). This analysis delineates the operational window and provides key parameters for power assessment.

**Figure 4:**
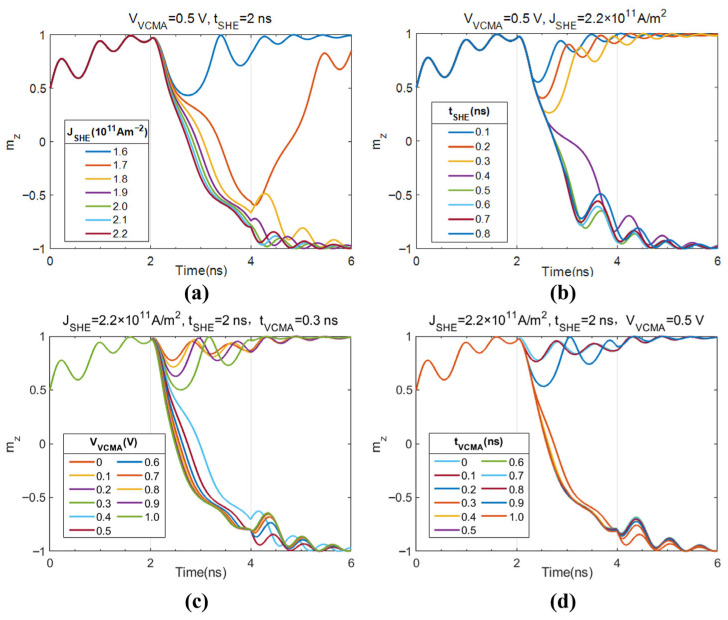
Effects of write current parameters and VCMA voltage on magnetization switching. (**a**) $m_{Z}$ versus HM current density; (**b**) $m_{Z}$ versus HM current pulse width; (**c**) $m_{Z}$ versus VCMA voltage amplitude; (**d**) $m_{Z}$ versus VCMA voltage pulse width.

To investigate the reduction in required write current density and pulse width for MTJ free layer magnetization switching with VCMA voltage assistance, the effect of varying write current on $m_{Z}$ change under a fixed VCMA voltage is plotted, as shown in Figure 4a,b. Here, the VCMA voltage amplitude is fixed at 0.5 V with a pulse width of 0.3 ns. Figure 4a shows the change in $m_{Z}$ under different $J_{SHE}$ amplitudes with a fixed SHE current pulse width. The critical current density is observed to be between $1.7\times 10^{11}A/M^{2}$ and $1.8\times 10^{11}A/M^{2}$. In Figure 4b, $J_{SHE}$ is fixed at $2.2\times 10^{11}A/M^{2}$. At this current density, a pulse width of at least 0.3 ns is required to complete the switch. Figure 4c,d plot the change in the magnetization z-component under different VCMA voltages. Here, the HM layer $J_{SHE}$ is fixed at $2.2\times 10^{11}A/M^{2}$ with a pulse width $t_{SHE}$ of 2 ns. When the VCMA pulse width is 0.3 ns, the change in $m_{Z}$ under different VCMA voltage amplitudes is shown in Figure 4c. It can be seen that VCMA voltages of less than 0.3 V and below are insufficient to cause successful switching of the MTJ free layer magnetization. Switching occurs with higher voltages, and larger VCMA voltages lead to faster switching. Figure 4d shows the change in $m_{Z}$ under different VCMA pulse widths with a fixed VCMA voltage amplitude of 0.5 V. It indicates that when the applied $t_{VCMA}$ is too narrow, the magnetization cannot complete the switch, requiring a pulse width of at least greater than 0.2 ns.

These results clearly map the “operational window” for VCMA-SHE cooperative switching. More importantly, they yield optimized parameters (e.g., $J_{SHE}=2.2\times 10^{11}A/M^{2}$, $t_{VCMA}=0.3NS$ and $t_{SHE}=2NS$), enabling a transition from qualitative description to a quantitative write power analysis. As summarized in Table 1, compared to a pure SOT-MTJ and STT-MTJ, the introduction of the VCMA effect effectively lowers the magnetic anisotropy barrier via a voltage pulse, substantially reducing the critical SOT current density required for switching. This directly leads to a significant reduction in Joule heating from the write path. Compared to STT-MTJ, our scheme retains the inherent reliability advantage of read/write path separation from the SOT mechanism while achieving markedly lower energy consumption via reduced operating current and shorter pulses.

The quantitative analysis demonstrates that our proposed VCMA-assisted SOT switching mechanism offers considerable improvements in two key metrics: write current density and write energy per bit. The achieved write energy of ~3.78 fJ/bit represents a drastic reduction compared to the typical ranges for STT and pure SOT schemes, respectively. This strongly affirms the potential of this hybrid mechanism for realizing high-efficiency, high-reliability spintronic memory and logic devices.

**Figure 5:**
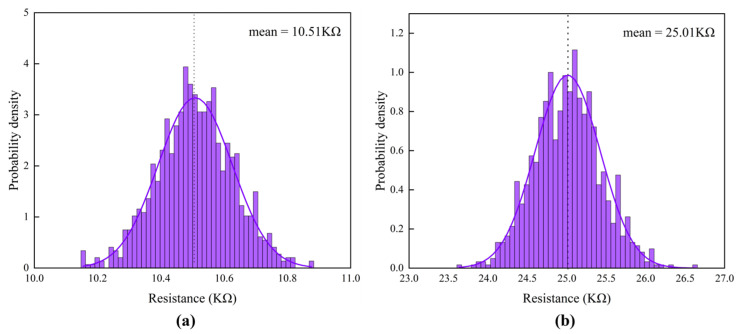
1000 times Monte Carlo results. (**a**) P→AP Switching: AP-State Resistance Distribution; (**b**) AP→P Switching: P-State Resistance Distribution.

Monte Carlo simulations of 1000 switching operations were performed to analyze the resistance distribution characteristics of SOT-MTJ devices during both P→AP and AP→P switching directions. As shown in Figure 5, the AP-state resistance during P→AP switching exhibits a normal distribution with a mean of 25.01 kΩ and standard deviation of 0.78 kΩ (CV = 3.12%). This relatively narrow distribution facilitates reduced read error rates and improved memory reliability. The calculated TMR of 137.96% falls within the typical range for SOT-MTJ devices (100–200%), indicating favorable magnetoresistive properties.

The observed resistance distribution standard deviations reflect the influence of actual process variations. The larger standard deviation for the AP state (0.78 kΩ) may originate from factors including magnetic tunnel junction area non-uniformity, barrier layer thickness fluctuations, and interface quality randomness. In contrast, the P-state resistance demonstrates lower sensitivity to process variations, consistent with theoretical predictions that electron tunneling in parallel magnetization configurations is less sensitive to barrier parameter changes.

In summary, the simulations reveal key characteristics of SOT-MTJ device resistance distributions: both states exhibit normal distributions with wider AP-state (σ = 0.78 kΩ) and narrower P-state (σ = 0.30 kΩ) distributions; the device demonstrates 137.96% TMR and a 14.50 kΩ resistance window, meeting basic requirements for non-volatile memory applications. These findings provide quantitative references for device design optimization.

### 2.4. Reliability and Endurance Analysis of the Device

Dielectric breakdown is a critical reliability challenge that spintronic devices must address in practical applications. When the insulating MgO layer in an MTJ is thin, an applied electric field can easily lower the potential barrier, leading to the formation of conductive filaments within the insulator and eventually causing a short circuit between the top and bottom electrodes. According to the dielectric breakdown model for CoFeB/MgO/CoFeB MTJs established by Wang et al. [31], the breakdown voltage $V_{B}$ can be expressed as a function of the barrier parameter $E_{B}$, the MgO thickness $t_{ox}$, and fitting parameters $V_{OFF}$ ($V_{B}=E_{B}\cdot t_{ox}+V_{OFF}$).

Based on the device parameters listed in Table 2, applying this model shows that under current fabrication conditions, both the forward and reverse breakdown voltages $V_{B}$ of the MTJ need to exceed 1.5 V. In the in-memory computing architecture proposed in this work, the VCMA voltage used to assist switching is strictly limited to below 1.5 V. Consequently, the operating voltage in our design remains well below the dielectric breakdown threshold, thereby preventing MTJ failure and ensuring long-term reliability. Moreover, the SOT mechanism inherently separates the read and write paths (read current flows vertically, write current flows within the heavy-metal layer), effectively eliminating read-disturb effects on the magnetization state and avoiding the tunneling-junction degradation commonly observed in STT-MRAM. This results in an endurance exceeding 10^14^ cycles.

## 3. Voltage-Controlled Stochastic Switching Device SHE-MTJ

With the rapid development of the digital industry, integrated circuits face increasingly stringent requirements for power consumption, area, and stability. Driven by novel computing architectures, memory devices, and diverse encoding techniques, the research focus is gradually shifting from traditional precise computing to stochastic computing paradigms. Stochastic computing performs operations using random bit streams, utilizing simple bit-level operations to achieve complex functions. It belongs to the category of approximate computing, offering advantages such as circuit simplicity, low power consumption, and strong fault tolerance.

MTJ devices inherently possess stochastic characteristics, mainly manifested in its random initial magnetization polar angle and its random thermal field $H_{\mathrm{th}ermal}$ caused by thermal fluctuations. Currently, the cutting-edge research contents in the field of spintronic devices include the random number generators, or stochastic generation and computation functions. In the section, starting from the stochastic LLG equation, the thermal fluctuation behavior of SHE-MTJs is simulated, and a voltage-controlled stochastic switching device can be achieved by modulating the switching probability via the VCMA effect.

The effect of thermal fluctuations on the magnetization of MTJ device is critical in the paper. In the standard LLG equation, the thermal fluctuations aren’t included. Thermal fluctuations not only affect the average precession behavior but also cause the initial magnetization angle $\theta_{0}$ to follow a random distribution. The initial angle $\theta_{0}$ directly affects the delay time of spin–current-driven magnetization switching. To simulate the MTJ initial magnetization angle influenced by thermal fluctuations, a random Gaussian distribution is used to represent $\theta_{0}$, with a mean of $\sqrt{1/2\Delta }$ and a variance of [9]:

(12) $$\langle \theta_{0}^{2}\rangle =\frac{k_{B}T}{M_{S}V\mu_{0}H_{k}},$$

Under the macrospin approximation, the random thermal field caused by thermal fluctuations can be expressed as:

(13) $$\begin{array}{l} \langle H_{\mathrm{thermal},i}\left(t\right)\rangle =0\\ \langle H_{thermal,i}\left(t\right)\cdot H_{thermal,j}\left(t+\Delta t\right)\rangle =\frac{2k_{b}T\alpha }{\mu_{0}\Omega }\delta_{i,j}\delta (\Delta t) \end{array},$$

where i, j represent subscripts for each of the two components in the Cartesian coordinate system, α is the Gilbert damping coefficient, Ω is the volume of the MTJ, $\delta_{i,j}$ is the Kronecker delta function in the i, j directions, and δ is the Dirac delta function. Equation (13) indicates that the random thermal field $H_{\mathrm{thermal}}$ is a stationary process, and the components of $H_{\mathrm{thermal}}$ follow a Gaussian distribution. The components are uncorrelated with each other, and the correlation time of the components is much shorter than the time required for the magnetization state to change [8].

Differentiating $H_{\mathrm{thermal}}$ over the time step dt yields the expression for the thermal fluctuation field:

(14) $$H_{\mathrm{thermal}}=G_{\left(0,1\right)}\sqrt{\frac{2\alpha k_{B}T}{\mu_{0}\gamma M_{S}\Omega dt}},$$

where $G_{\left(0,1\right)}$ is a standard Gaussian distribution.

**Figure 6:**
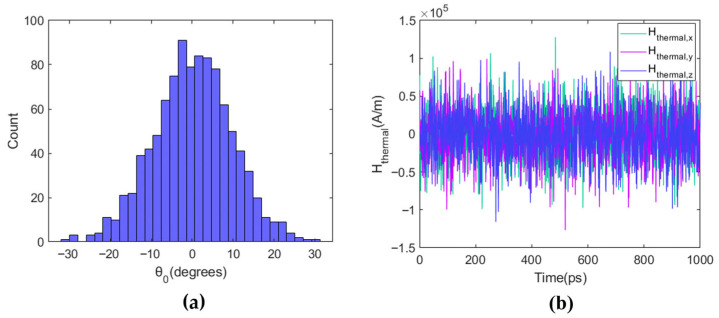
Initial angle distribution and stochastic thermal field due to thermal fluctuations. (**a**) Statistical distribution of 1000 random initial magnetization angles ($\theta_{0}$); (**b**) Instantaneous variations of the three components of the thermal fluctuation field $H_{\mathrm{thermal}}$ within 1 ns.

Figure 6a shows the distribution of 1000 random initial magnetization angles $\theta_{0}$ based on Equation (14). It can be seen that for the same MTJ, the variation in $\theta_{0}$ distribution is considerable. Under otherwise identical conditions, larger $\theta_{0}$ leads to a shorter switching time required for the MTJ. Figure 6b shows the variation of the three components of the thermal fluctuation field $H_{\mathrm{thermal}}$ within a 1 ns simulation time. To study the stochastic device for accelerating computing, the model parameters used in this research are listed in Table 2. The selection of key physical parameters was based on published experimental and theoretical literature [17,18,19].

**Figure 7:**
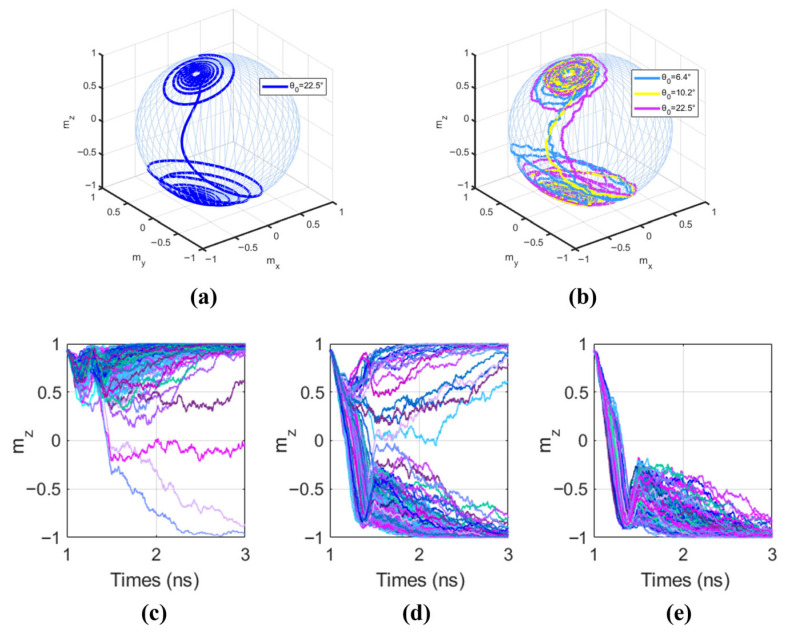
Impact of thermal fluctuations on magnetization precessiosn and switching probability. (**a**) Magnetization precession without thermal fluctuations ($\theta_{0}$ = 22.5°); (**b**) Comparison of magnetization precession under thermal fluctuations with different initial angles; (**c**–**e**) Statistical evolution trajectories of $m_{Z}$ under different VCMA voltages: (**c**) $V_{VCMA}$ = −0.35 V, (**d**) $V_{VCMA}$ = 0.05 V, (**e**) $V_{VCMA}$ = 0.35 V.

To observe the effect of thermal fluctuations on magnetization, the precession behavior of magnetization with/without the thermal field is compared, as shown in Figure 7. Figure 7a shows the precession of magnetization successfully switching under the drive of a 0.5 V VCMA voltage and a $2.2\times 10^{11}A/M^{2}$ HM layer current, without considering thermal fluctuations and with an initial magnetization angle $\theta_{0}$ of 22.5°. Figure 7b shows the precession behavior under the influence of thermal fluctuations for three different initial magnetization angles under the same voltage and current drive. It can be seen that magnetization with a larger initial angle switches faster.

Figure 7c,d plots the different evolution trajectories of the MTJ free layer magnetization $m_{Z}$ component under the influence of random thermal fields when applying different VCMA voltages with $J_{SHE}$ fixed at $2.2\times 10^{11}A/M^{2}$. Observing Figure 7c, when the VCMA voltage is −0.35 V, the MTJ energy barrier is increased, and the magnetization can hardly be switched. However, due to the presence of random thermal fluctuations, a very small number of magnetization vectors can overcome the damping torque with the assistance of $H_{\mathrm{thermal}}$, showing the tendency of precess downward, and eventually switch successfully. Figure 7d shows the case with a VCMA voltage of 0.05 V. With VCMA voltage assistance, most MTJ magnetizations complete the switch, while a minority, influenced by $H_{\mathrm{thermal}}$, oscillate back to the initial state and fail to switch. Figure 7e shows the case with a VCMA voltage of 0.35 V. When the voltage applied to the MTJ is sufficiently large, the influence of $H_{\mathrm{thermal}}$ is eliminated. It can be observed that all magnetization vectors successfully complete the switch. After the voltage and current stop, the magnetization precesses and gradually stabilizes at the −1 state.

**Figure 8:**
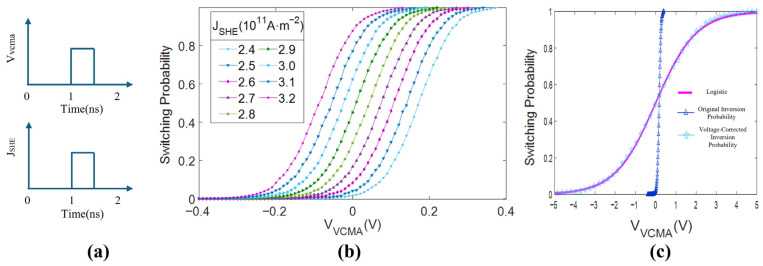
Characteristics of the voltage-controlled stochastic switching SHE-MTJ. (**a**) Timing of applied VCMA voltage and HM current pulses; (**b**) Switching probability versus VCMA voltage under different HM current densities; (**c**) Comparison between measured data and Logistic fitting.

Due to the presence of the random thermal fluctuation field $H_{\mathrm{thermal}}$, different magnetization switching trajectories inevitably occur. Simulations are conducted 10^3^ simulations for each combination of VCMA voltage and heavy metal current density. In the simulations, the VCMA voltage and HM layer current are applied simultaneously at 1 ns. After a pulse width of 0.5 ns, they are stopped simultaneously at 1.5 ns, which corresponds to approximately half a precession period, as shown in Figure 8a. The simulation then continues until reaching a steady state, where $m_{Z}$ attains either +1 or −1 for each trajectory.

The number of successfully switched MTJs is counted and organized for different $V_{VCMA}$ and $J_{SHE}$ conditions. The z-component of the magnetization vector $m_{Z}$ is plotted for each case, as shown in Figure 8b. It can be observed that the switching probability curves as a function of VCMA voltage exhibit a nearly perfect “S” shape. As $J_{SHE}$ varies from $2.4\times 10^{11}A/M^{2}$ to $3.2\times 10^{11}A/M^{2}$, the shape of the curves does not change significantly but undergoes a certain shift. This near-perfect probabilistic switching characteristic is highly suitable for accelerating probabilistic computations. As shown in Figure 8c, one switching curve ($J_{SHE}=2.4\times 10^{11}A/M^{2}$) is chosen and fitted with a sigmoid curve. After applying the obtained bias and scaling factor for translation and scaling, it aligns almost perfectly with the sigmoid function.

For the switching probability curves under different $J_{SHE}$ values, different voltage parameters (bias $V_{bias}$ and scaling factor $V_{0}$) can be added to adjust the MTJ device to achieve a sigmoidal switching probability as a function of VCMA voltage:

(15) $$Sig\mathrm{mod}\left(\frac{V_{VCMA}-V_{bias}}{V_{0}}\right)\approx SwitchingP\left(V_{VCMA}\right),$$

where $V_{bias}$ is the bias used to adjust the MTJ switching probability to be 0.5 when VCMA voltage is 0, and $V_{0}$ is the scaling factor that extends the switching curve to match the sigmoid swing, facilitating subsequent neuron circuit design.

## 4. Binarized SqueezeNet

Aiming at the convolutional neural networks (CNNs) widely used in current image classification and object detection tasks, an in-memory computing acceleration scheme based on MTJ crossbar arrays is proposed in the study. Utilizing the aforementioned voltage-controlled SHE-MTJ with stochastic switching characteristics to construct neurons, it simultaneously achieves binarized input (XNOR operation) and introduces stochasticity, making it closer to biological neuron characteristics and helping prevent overfitting. Considering the area, speed, power consumption, and cost of on-chip acceleration, the lightweight network SqueezeNet is adopted as the basis for structural adjustment and validates the scheme’s feasibility on the CIFAR-10 dataset [32,33].

**Figure 9:**
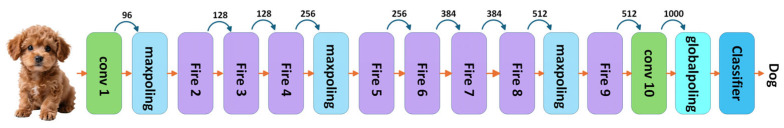
Overall architecture of the lightweight CNN, SqueezeNet.

Figure 9 shows the lightweight SqueezeNet network structure adapted for in-memory computing acceleration. The input image is processed by convolution layer 1, downsampled by a max pooling layer, sequentially fed into three stacked Fire modules, pooled again, fed into four Fire modules, and finally passed through one more Fire module and a convolution layer to extract features. The output is generated via global pooling and a classifier.

**Figure 10:**
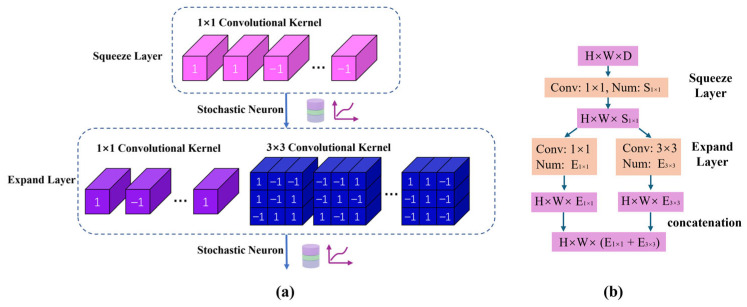
Modified Fire module structure for in-memory computing. (**a**) Schematic of the module; (**b**) Internal operations and feature dimensions.

**Figure 11:**
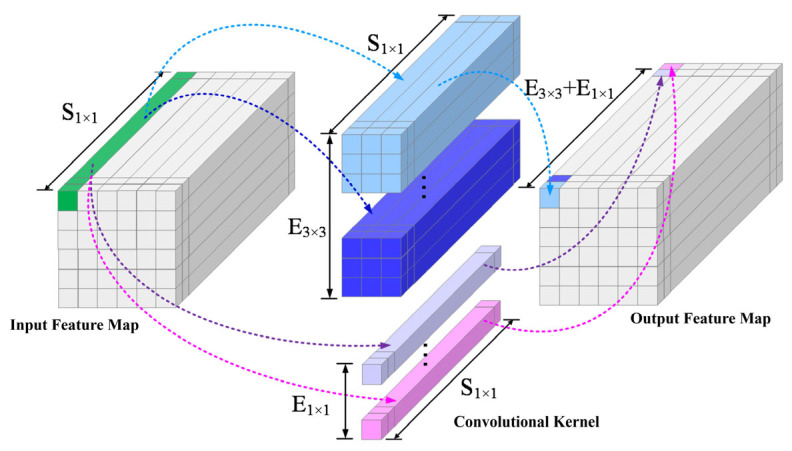
Dataflow of convolutional computation in the Expand layer of a Fire module.

Figure 10 presents the modified Fire module structure to adapt to MTJ in-memory computing, with Figure 10b showing the details of its internal operations, kernel counts, and output dimensions. Each Fire module consists of a Squeeze layer and an Expand layer. The Squeeze layer contains $S_{1\times 1}$ 1 × 1 convolution kernels, significantly reducing the parameter count and the number of input channels for the next layer through small-sized convolutions. To support in-memory computing, the kernel weights are binarized to +1/−1. The output features $H\times W\times S_{1\times 1}$ from the Squeeze layer are sent to stochastic neurons. Their probabilistic switching results serve as the input for the Expand layer. The Expand layer contains $E_{1\times 1}$ 1 × 1 kernels and $E_{3\times 3}$ 3 × 3 kernels. After separately convolving the input, the output features are processed by stochastic neurons and then concatenated along the depth dimension, resulting in output feature maps of size $H\times W\times (E_{1\times 1}+E_{3\times 3})$. Using the Expand layer as an example, Figure 11 illustrates the data flow of the convolution operation: the input feature map has a depth of $S_{1\times 1}$, which is convolved with the 1 × 1 and 3 × 3 kernels separately. The results are concatenated to output feature maps with a depth of $\left(E_{1\times 1}+E_{3\times 3}\right)$.

## 5. In-Memory Computing Circuit

**Figure 12:**
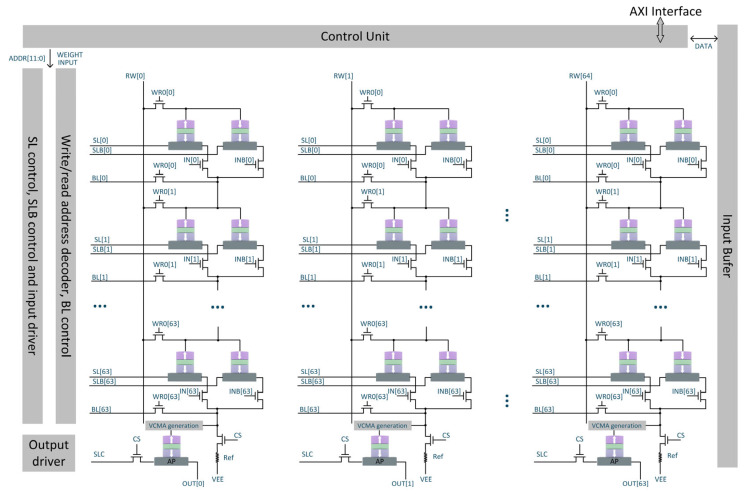
SHE-MTJ-based stochastic neuron and its crossbar array architecture.

Biological neurons possess stochastic characteristics, meaning under identical stimulus inputs, different neurons may have slightly different outputs. To mimic the stochasticity of biological neurons, the voltage-controlled stochastic switching SHE-MTJ is used to build stochastic neurons suitable for binarized convolutional neural networks, as shown in Figure 12. The SOT-MTJ CIM consists of a 64 × 64 array, in which each cell is composed of a pair of complementary SOT-MTJ devices. Input data and its inverted counterpart are fed into the array through the IN and INB lines, respectively. The writing and computing operations of the MTJ are controlled coordinately by multiple signal lines, including RW, WR, SL, SLB, BL, IN, and INB. The equivalent column resistance $R_{eff}$ of each MTJ crossbar array is divided with a reference resistor $R_{ref}$. The voltage at the $V_{VCMA}$ node serves as the VCMA voltage for the SHE-MTJ to control its switching probability. After the operation, the resistance state of the MTJ within this neuron is detected as the output. If the divided voltage is insufficient, sampling according to the stochastic switching probability curve yields 0 (neuron did not switch), and the output is the $R_{AP}$ value (high resistance). If the divided voltage is sufficiently large, sampling yields 1 (neuron switched), and the output is the $R_{P}$ value (low resistance). Figure 12 shows a schematic of the convolutional crossbar array structure. The convolution operation results from the crossbar array become the VCMA voltages for the neurons in the current layer. In XNOR-Net, each binarized convolution layer operation essentially involves sliding binarized (“1” and “−1”) kernels over the binarized (“1” and “−1”) input feature maps. Inputs are binarized using the sign function, but points with a value of 0 are also set to “−1”.

**Figure 13:**
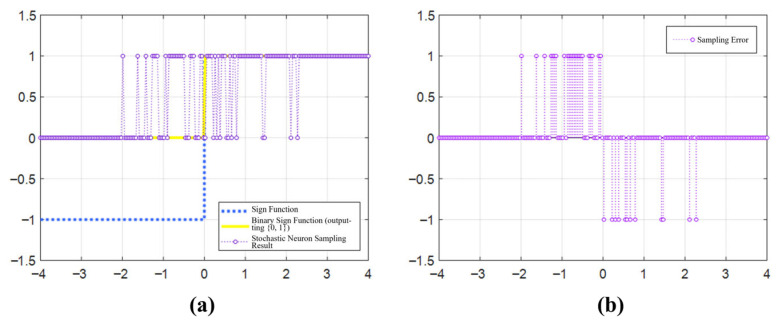
Response characteristics and error analysis of the stochastic neuron. (**a**) Comparison between stochastic sampling outputs and the ideal sign function; (**b**) Distribution of the introduced sampling error.

In this study, we represent the “±1” of the sign function using the “01” states of the MTJs used. According to the different VCMA voltages assigned to each column of MTJs, the MTJs undergo stochastic switching with the probability shown in Figure 8c. The sampling results are illustrated in Figure 13a. When a neuron receives a very large positive voltage, it almost certainly switches. On the contrary, when it receives a very large negative voltage, it almost certainly does not switch. When the voltage is around 0, the neuron has a higher probability of not flipping according to the sign function. Figure 13b plots the sampling error of the stochastic neuron. By introducing a certain probability of error into the SqueezeNet neurons, overfitting during network training with small image samples can be prevented, while the network’s classification capability for prediction samples can be enhanced.

**Figure 14:**
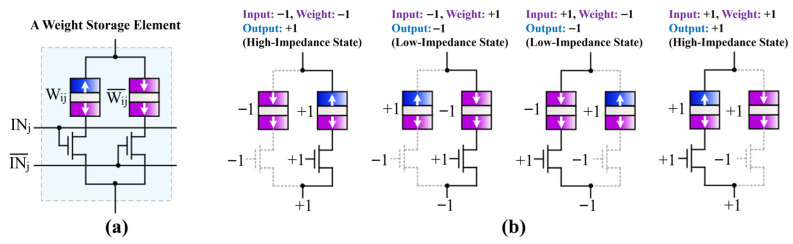
Binary weight storage cell structure and XNOR operation truth table. (**a**) Dual-MTJ-based weight storage cell; (**b**) Four I/O configurations of the XNOR operation.

To achieve CNN acceleration with the MTJ array, during the training process, all convolutional layers in the SqueezeNet Fire modules are binarized. This allows the input of each Fire module layer to be represented by MTJ neuron sampling, and each convolution kernel can be stored by an MTJ array. Because both the high and low resistances of MTJs are relatively small and the convolutional network scale is large, using the traditional current-sum mode for convolution operations would undoubtedly consume considerable power [2]. Therefore, the crossbar array in this study adopts a resistance-sum mode to obtain the multiply-accumulate sum result. Figure 14a shows a schematic of the MTJ storage cell for weight $W_{ij}$, corresponding to each MTJ-Cell in Figure 11. Each weight storage cell consists of two MTJs representing $W_{ij}$ and $\bar{W_{ij}}$, and two MOS transistors controlled by inputs $IN_{j}$ and $\bar{IN_{j}}$. The MTJ representing $W_{ij}$ is connected to the MOS transistor controlled by $IN_{j}$, and the MTJ representing $\bar{W_{ij}}$ is connected to the MOS transistor controlled by $\bar{IN_{j}}$. Since both inputs and weights are “±1” in XNOR-Net, the convolution operation is equivalent to an XNOR operation. Figure 14b describes the four different input-output configurations for the XNOR operation during convolution. When the input is “−1”, the left MOS transistor connected to the MTJ representing $W_{ij}$ is turned off, and the right MOS transistor connected to the MTJ representing $\bar{W_{ij}}$ is turned on. The output resistance of this storage cell is the resistance of the MTJ representing $\bar{W_{ij}}$. When this weight is “−1”, the output resistance corresponds to “+1” (high resistance). When this weight is “+1”, the output resistance corresponds to “−1” (low resistance). Thus, the IN⊙W operation is completed. The cases for other inputs are similar.

The resistance sum $R_{eff}$ of each column of storage cells corresponds to the multiply-accumulate sum result of the binarized input and binarized weights, which is also the input for the neuron connected to that column. This $R_{eff}$ forms a voltage divider with $R_{ref}$ in the neuron structure shown in Figure 12. The voltage at the $V_{VCMA}$ node is the switching control voltage for the MTJ. When the output result of the column is larger, the voltage at $V_{VCMA}$ is smaller, and the probability of the MTJ switching is lower. The unswitched MTJ resistance $R_{AP}$ (high resistance) is the neuron’s output resistance, representing “1”. When the column’s output result is smaller, the voltage at $V_{VCMA}$ is larger, and the probability of the MTJ switching is higher. The switched MTJ resistance $R_{P}$ (low resistance) is the neuron’s output resistance, representing “0”. Here, the MTJ neuron implements both the binarization function of the sign function and introduces input-dependent stochasticity, making it more akin to biological neuron characteristics.

**Figure 15:**
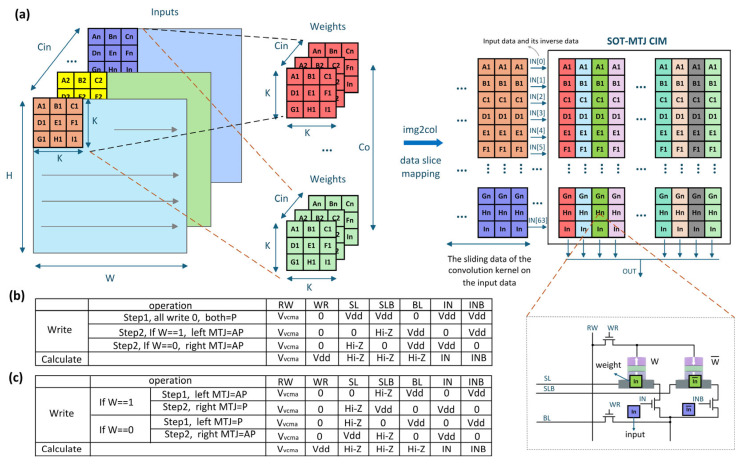
A schematic of the convolution operation and the corresponding writing schemes. (**a**) The convolution process, where the input feature map is operated via a kernel mapped into the SOT-MTJ array. (**b**) Writing scheme 1: all cells are first written to “0,” followed by selective writing of “1.” (**c**) Writing scheme 2: selective writing of both “1” and “0” is performed directly.

For the convolution operation, weight data are initially written into the memory array via the AXI protocol. Two writing schemes are proposed, as shown in Figure 15b,c. The first scheme requires writing all cells to “0” before selectively writing “1” under logical control of each line voltage. The second scheme enables selective writing of “0” and “1” directly through the controller. Simulation based on a 65 nm technology library demonstrates that the second scheme improves energy efficiency by 22% compared with the first scheme. Therefore, the second scheme is adopted as the writing strategy in this design. The input features are reconstructed via an image-to-column (im2col) transformation to facilitate efficient binary matrix-vector multiplication (BMVM) using the kernel weights, as shown in Figure 15a. Input data are first written into a buffer adjacent to the memory array through the im2col module. The controller then fetches the data and computes its complement, which is subsequently fed into the memory array in a serial manner under a 33 MHz clock cycle.

Parasitic resistance in the interconnects of large-scale computing-in-memory arrays is a critical factor influencing computational accuracy and system feasibility. This study presents a systematic analysis of parasitic effects in a 64 × 64 resistive memory crossbar array implemented in 65 nm technology. Each column of memristors is connected in series with a reference resistor to form a voltage divider network, enabling analog current summation. The parasitic resistance originates mainly from the bulk resistance of metal interconnects, distributed RC effects, and interlayer contact resistance. By extracting the interconnect electrical parameters of this technology node and constructing a distributed RC model, we estimate that the total line parasitic resistance per column is approximately 660 Ω. Incorporating this parasitic resistance into the voltage divider model shows that across the dynamic range of the column resistance (672.64 kΩ to 1.60 MΩ), the maximum absolute output voltage deviation caused by parasitic effects is on the order of 2 μV, with a relative error below 0.0005%. This deviation is significantly smaller than the quantization step of typical converters in readout circuits and is masked by the inherent circuit noise, thus having a negligible impact on the accuracy of individual analog multiply-accumulate operations. At the system level, neural networks exhibit inherent robustness to minor weight perturbations. Our analysis indicates that the equivalent noise introduced by these parasitic effects is well below the error tolerance of standard quantized model training, and thus is not expected to significantly degrade final inference accuracy.

## 6. Results

The experimental validation on the CIFAR-10 dataset is conducted in the paper. The training batch size is set to be 256 for a total of 150 epochs, using the Stochastic Gradient Descent algorithm with a momentum of 0.9. To balance convergence speed in the early stages and stability in the later stages, a piecewise constant learning rate decay strategy is adopted, with the reduction of the learning rate of 0.1 times its previous value at epochs 80, 100, 120, and 140.

**Figure 16:**
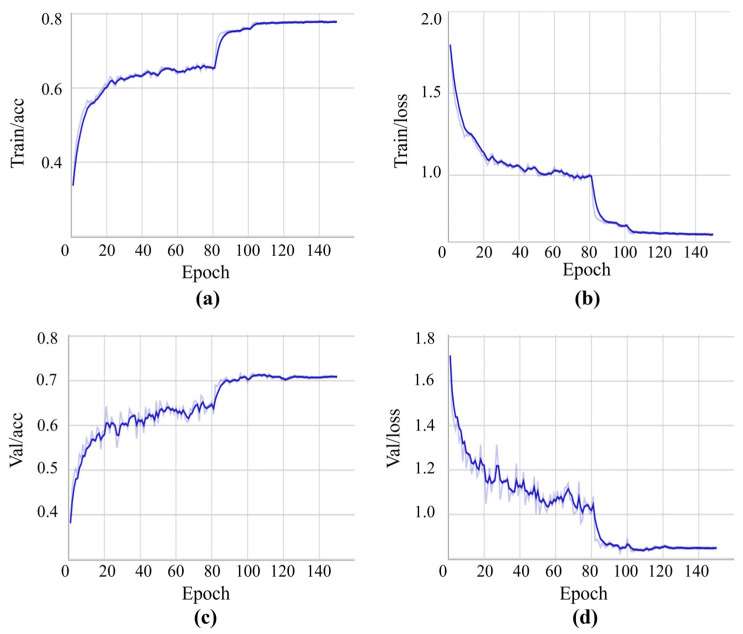
Training and validation curves of the binarized SqueezeNet on CIFAR-10. (**a**) Training Top-1 accuracy; (**b**) Training loss; (**c**) Validation Top-1 accuracy; (**d**) Validation loss.

Figure 16 shows the training process of the binarized SqueezeNet. Figure 16a shows the Top-1 accuracy on the training set, and Figure 16b shows the training loss curve. The network converges smoothly, with the convergence rate slowing down after approximately epoch 30 and saturating after epoch 60. After the learning rate decay at epoch 80, the network continues to converge. Subsequent decays are also effective, with optimal performance achieved and stabilized after epoch 120. The final training set accuracy reached 78.98%. Figure 16c and Figure 16d show the validation set accuracy and loss curves, respectively. On the test set, the network achieved a Top-1 accuracy of 72.49% and a Top-5 accuracy of 97.75%.

**Figure 17:**
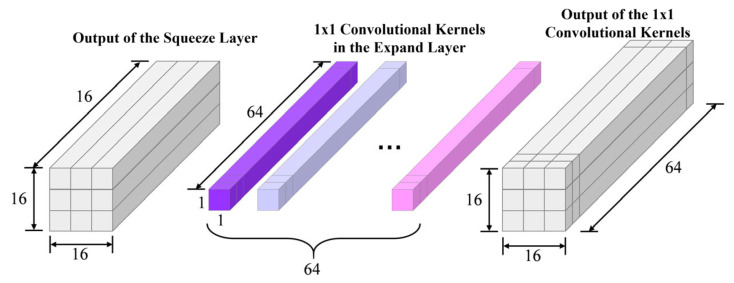
Schematic of the convolutional computation flow in the Expand layer of a Fire module.

**Figure 18:**
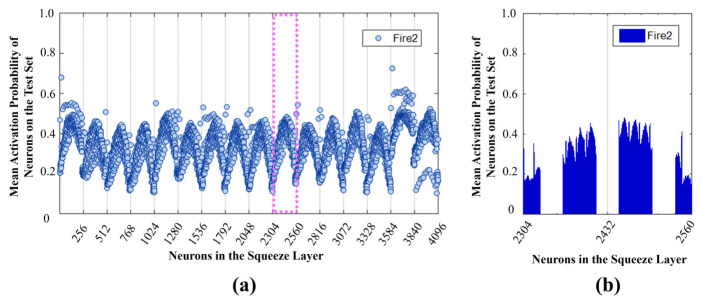
Average activation probability of neurons in the Squeeze layer during testing. (**a**) Average activation probability of all 4096 neurons; (**b**) Activation probability map of a single feature map (16 × 16).

**Figure 19:**
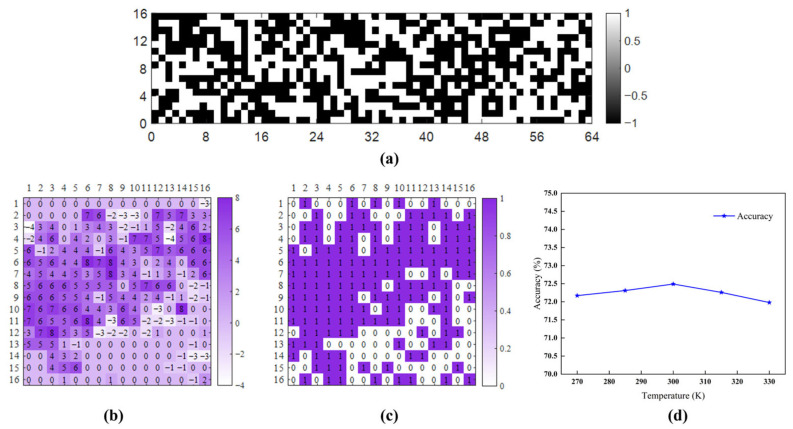
Weight distribution and neuronal activity analysis in the Expand layer of Fire module 2. (**a**) Visualization of binary weights in 64 1 × 1 kernels; (**b**) Analog input values to a subset of neurons; (**c**) Corresponding stochastic switching outputs; (**d**) The impact of temperature changes on accuracy.

To analyze the behavior of the SHE-MTJ stochastic neurons, Fire module 2 is taken as an example. This module first converts the input into 16 × 16 × 16 feature maps through the Squeeze convolution layer. Subsequently, in the Expand layer, convolution is performed with 1 × 1 and 3 × 3 kernels. The Expand layer contains 64 kernels of size 1 × 1, each with a depth of 16. After convolution, it outputs 16 × 16 × 64 feature maps, which are then concatenated with the output from the 3 × 3 convolution. The following analysis focuses on the 1 × 1 convolution operation, as shown in Figure 17. 2000 images are randomly selected from the test set and fed into the trained network. The activation count of each output neuron in the Squeeze layer of Fire module 2 for each image is recorded, and the average activation probability is calculated. The results are shown in Figure 18a. The Squeeze layer has a total of 4096 output neurons. Every 16 × 16 neurons correspond to one feature map, totaling 16 feature maps. The figure shows that neuron activation probabilities exhibit clear grouping, with each group corresponding to different feature extraction patterns. Within each group, neurons at the corners of the feature maps have lower activation probabilities, indicating weaker feature saliency in these regions. Figure 18b shows the detailed activation probability distribution for one group (neuron indices 2304–2560). It can be seen that within the 16 × 16 feature map, there is still a grouping phenomenon between rows, and edge neurons generally have lower activation probabilities. Figure 19a shows the distribution of binarized weights for the 1 × 1 kernels in the Expand layer of Fire module 2. There are 64 kernels of size 1 × 1 × 16. White represents −1, and black represents +1. This distribution corresponds to the actual weight configuration in the MTJ crossbar array.

The Expand layer’s 1 × 1 convolution outputs 64 feature maps of size 16 × 16. One of these feature maps is selected to analyze the neuron inputs and stochastic switching results. Figure 19b shows the input value for each neuron obtained through the crossbar array resistance sum, and Figure 19c shows the corresponding stochastic sampling output of the neurons. Statistics show that the binarization error rate introduced by the stochastic neurons here is 14.45%. The errors are mainly distributed in regions where the input values are close to zero, therefore having a limited impact on the overall network performance. Simultaneously, they effectively help suppress overfitting in the in-memory computing architecture. However, the lower validation set accuracy compared to the training set indicates that the current error rate is still slightly high, affecting the model’s predictive performance to some extent.

To assess the temperature tolerance of the proposed scheme, classification tests were performed at different temperatures. Figure 19d plots the variation in classification accuracy for a 64 × 64 array across temperature. As shown in Figure 19d, the model’s accuracy changes only minimally within a temperature range fluctuating around 300 K. This indicates two important points: First, although the switching probability curve of the physical device shifts with temperature, this shift is uniform across the operating range. Second, the resistance-weighted in-memory computing scheme adopted in this work, together with the binarized neural network itself, exhibits strong immunity to temperature variations. Furthermore, in our simulation tests, a noise distribution (Monte Carlo simulations) was inherently incorporated into the resistance states of the MTJs, effectively emulating column-resistance variations due to process fluctuations. The results demonstrate that the network maintains excellent tolerance to these disturbances.

Model parameter statistics show that the modified SqueezeNet used in this scheme contains a total of 1,245,544 parameters, achieving a Top-1 recognition accuracy of 72.49% (Top-5 97.75%) on CIFAR-10. The original SqueezeNet has 1,248,424 parameters and a Top-1 accuracy of 86.72%. Compared to the original network, this scheme incurs some accuracy loss due to binarization and stochastic neurons, but the parameter count is slightly reduced. More importantly, the characteristics of in-memory computing—parallel processing, data processing in place, resistance summation—fundamentally reduce power consumption and increase operational speed, demonstrating significant research value and application potential.

**Figure 20:**
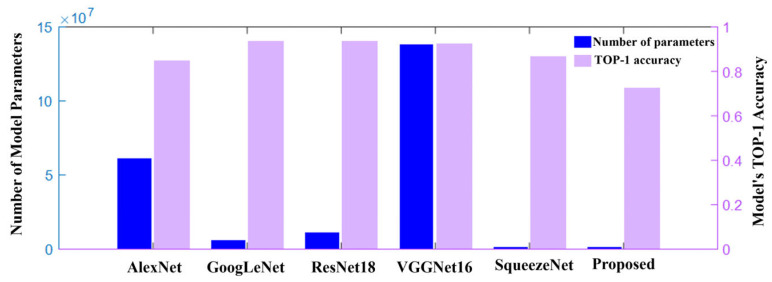
Comparison of parameter count and Top-1 accuracy among mainstream CNN models on CIFAR-10.

Figure 20 compares the parameter counts and Top-1 accuracy of mainstream image recognition models on CIFAR-10. AlexNet has about 6.1 × 10^7^ parameters and an accuracy of 84.91%. GoogLeNet and ResNet-18 significantly reduce parameters while maintaining high accuracy (>93%) through special module structures. VGG-16 has an extremely high parameter count due to its deep structure, numerous kernels, and large fully connected layers. In contrast, SqueezeNet and our scheme maintain respectable accuracy with significantly reduced parameter counts, making them more suitable for edge-side intelligent chip application scenarios.

As shown in Table 3, a key advantage of the proposed scheme over recently reported in-memory computing architectures is that it does not require costly analog-to-digital converters (ADCs) or time-to-digital converters (TDCs). It should be noted that while the accuracy values are listed for reference, a direct comparison is limited by differences in neural network models and datasets across the cited works. Conventional resistive-device-based in-memory computing typically employs a current-summation scheme, which relies on high-precision ADCs to convert analog currents into digital signals—a process that consumes significant static power. In contrast, the architecture presented here utilizes a resistive voltage-divider network to directly convert analog multiply-accumulate results into the voltage that drives the next layer of MTJ neurons (i.e., the probability-control voltage). This fully-analog/mixed-signal approach entirely eliminates the area and energy overhead associated with ADCs and TDCs. Combined with the extremely low-power VCMA-assisted switching MTJ devices, the scheme thereby achieves higher overall energy efficiency.

## 7. Conclusions

In the paper, VCMA effect in MTJ devices is investigated, proposing a novel device that utilizes VCMA to assist SHE for MTJ switching. A macrospin model is established based on the LLG equation, and its dynamic characteristics are analyzed. It is shown that applying VCMA voltage pulses with appropriate amplitude and width during SHE writing can significantly reduce the required HM layer write current density and pulse width, effectively minimizing ohmic losses and Joule heating. Furthermore, by incorporating a thermal fluctuation field, a voltage-controlled stochastic switching SHE-MTJ device is constructed, achieving a voltage-probability response curve close to an ideal sigmoidal shape. This provides a device foundation for stochastic computing and AI acceleration.

A neuron combining binarization and stochastic activation functions is designed for CNNs based on this stochastic switching device. An in-memory computing acceleration scheme for the lightweight network SqueezeNet is proposed. Binarized weights are stored in MTJ crossbar arrays, XNOR multiply-accumulate operations are realized through resistance summation, and the SHE-MTJ neurons perform the sign function mapping with stochasticity. This scheme achieved a Top-1 accuracy of 72.49% and a Top-5 accuracy of 97.75% on the CIFAR-10 dataset, with a parameter count of only 1.25 × 10^6^. Compared to the traditional von Neumann architecture, this scheme effectively reduces data movement overhead through in-memory computing, making it more suitable for low-power intelligent processing scenarios at the edge. It provides valuable reference for the design and implementation of spintronic in-memory computing chips.

## Figures and Tables

**Table 1:** Performance comparison of different MTJ writing technologies.

	STT-MTJ	SOT-MTJ	VCMA-SOT-MTJ (This Work)
Switching Mechanism	Spin–polarized current	SOT from in-plane current in HM	VCMA modulation + SOT driving
Read/Write Path Separation	No	Yes	Yes
Cell area	√	√√	√√
Write Latency	<10 ns	<3 ns	2 ns
Read Latency	<10 ns	~1 ns	0.5 ns
Write Energy	0.1~1 pJ	0.3~1.8 pJ	3.78 fJ
Endurance	1 × 10^6^~1 × 10^14^	~1 × 10^12^	~1 × 10^14^

**Table 2:** Stochastic SHE-MTJ Model Parameter Definition.

Parameter	Description	Value
K_s0_	Interface anisotropy energy density	320 μJ/m^2^
M_s_	Saturation magnetization	6.25 × 10^5^ A/m
κ	Voltage-controlled anisotropy coefficient	50 fJ V^−1^ m^−1^
d	MTJ diameter	50 nm
t_ox_	Tunnel barrier thickness	1.5 nm
t_f_	Free layer thickness	1.1 nm
L	Heavy metal layer length	100 nm
w	Heavy metal layer width	100 nm
h	Heavy metal layer thickness	3 nm
η	Spin Hall angle	0.3
ρ_HM_	Heavy metal layer resistivity	200 μΩ·cm
T	Temperature	300 K
H_external_	External magnetic field	4.8 × 10^4^ A/m
α	Gilbert damping constant	0.05

**Table 3:** Comparison with recent Cim Works.

	JSSCC’24 [34]	VLSI’25 [35]	Nature’22 [2]	This Work
Technology	65 nm	65 nm	28 nm	**65 nm**
Synapse	1T1C/eDRAM	2T2R/RRAM	2T2R/STT-MTJ	**4T2R/VCMA-SOT-MTJ**
Nonvolatility	No	Yes	Yes	**Yes**
Capacity	16 Kb	8 Kb	4 Kb	**4 Kb**
Activations/Weigths	8b/8b	1b/1b	1b/1b	**1b/1b**
With ADC/TDC	Yes	No	Yes	**No**
Frequency	N/A	136 MHz	11.1 MHz	**33 MHz**
Power Consumption	N/A	0.625 mW	0.225 mW	**0.177 mW**
Peak Energy Efficiency	4.76 TOPS/W	578 TOPS/W	405 TOPS/W	**1527 TOP/W**
Network Supported	MLP/CNN	CNN	MLP	**MLP/CNN**
Accuracy	80.1%	90.07%	93.4%	**72.49%**

## Data Availability

The original contributions presented in this study are included in the article. Further inquiries can be directed to the corresponding author.
